# Characterization of Organic Acid Metabolism and Expression of Related Genes During Fruit Development of *Actinidia eriantha* ‘Ganmi 6’

**DOI:** 10.3390/plants9030332

**Published:** 2020-03-05

**Authors:** Zhiqiang Jiang, Qing Huang, Dongfeng Jia, Min Zhong, Junjie Tao, Guanglian Liao, Chunhui Huang, Xiaobiao Xu

**Affiliations:** Institute of Kiwifruit, Jiangxi Agricultural University, Nanchang 330045, China; 13870113504@163.com (Z.J.); huangqing9293@163.com (Q.H.); dongfengjia@163.com (D.J.); chenmined@126.com (M.Z.); taojj@jxau.edu.cn (J.T.); liaoguanglian@163.com (G.L.)

**Keywords:** *Actinidia eriantha*, ‘Ganmi 6’, organic acid, enzyme activity, gene expression

## Abstract

Studies on organic acid metabolism have been mainly concentrated on the fruit, whereas, few have focused on the mechanism of high organic acids content in the fruit of *Actinidia eriantha*. Fruits of ‘Ganmi 6’ harvested at eleven developmental periods were used as materials. The components and content of organic acids were determined by high-performance liquid chromatography (HPLC) system, the activities of the related enzyme were detected, and gene expression levels were measured by quantitative real-time PCR (qRT-PCR). Components of ascorbic acid (AsA) and eight kinds of organic acids were detected. These results showed that quinic acid and citric acid were the main organic acids in the fruit of ‘Ganmi 6’. Correlation analysis showed that NADP-Quinate dehydrogenase (NADP-QDH), NADP-Shikimate dehydrogenase (NADP-SDH), and Cyt-Aconitase (Cyt-Aco) may be involved in regulating organic acids biosynthesis. Meanwhile, the *SDH* gene may play an important role in regulating the accumulation of citric acid. In this study, the activities of NADP-SDH, Mit-Aconitase (Mit-Aco), and NAD-Isocitrate dehydrogenase (NAD-IDH) were regulated by their corresponding genes at the transcriptional level. The activity of Citrate synthase (CS) may be affected by post-translational modification. Our results provided new insight into the characteristics of organic acid metabolism in the fruit of *A. eriantha*.

## 1. Introduction

China is the origin and distribution center of kiwifruit. It has abundant germplasm resources of kiwifruit [1]. Kiwifruit is rich in organic acids, sugars, mineral elements, and other nutrients, which play important roles in human health [2]. *Actinidia eriantha* belongs to the family Actinidiaceae, genus *Actinidia*, which is a unique kiwifruit germplasm resource in China [3]. *A. eriantha* has attracted much attention, due to its green flesh, strong fruit flavor, high ascorbic acid (AsA) content, easy peeling and ornamental value of pink corolla [4]. ‘Ganmi 6’ is a new kiwifruit cultivar which was selected from wild *A. eriantha* in Yihuang County, Jiangxi Province, China by Institute of kiwifruit, Jiangxi Agricultural University. The fruit shape is long cylindrical with short white hairs, the average fruit weight is 72.5 g, and the maximum is 96.0 g. The soluble solids content in fruit is 13.6%, titrable acid is 0.87%, and the dry matter is 17.3%. The ripening fruit is easily peeling, AsA content is 7.23 mg·g^−1^ fresh weight (FW), and it matures in late October [5].

Organic acid are one of the major class of phytochemicals in fruits and responsible for food taste and odor, and play important roles in maintaining fruit quality and nutritional value. The variations of organic acids content in different fruit developmental periods vary drastically among species [6,7,8]. There are many kinds of organic acids in the fruit, but most of the fruits are mainly composed of one or two main organic acids. Three major organic acids were accumulated in most fruits, including malic acid, citric acid, and tartaric acid [9]. Different kinds of fruits contain different organic acid. For instance, citric acid is the predominant organic acid in the fruits of *Citrus* [10], the major organic acid in apple fruit is malic acid [9], while the main organic acid in grape fruit is tartaric acid [11]. Previous studies showed that many kinds of organic acids components had been found in kiwifruit of different genotypes [12,13,14]. Quinic acid was considered the main organic acid in the fruit of ‘Hayward’ [15,16]. However, some studies reported that citric acid, malic acid and quinic acid were the major organic acids in the fruits of ‘Hayward’ and ‘Bruno’ that are grown in New Zealand [17,18]. However, scientific information on organic acids components and content in the fruit of *A. eriantha* is still unknown.

**Figure 1:**
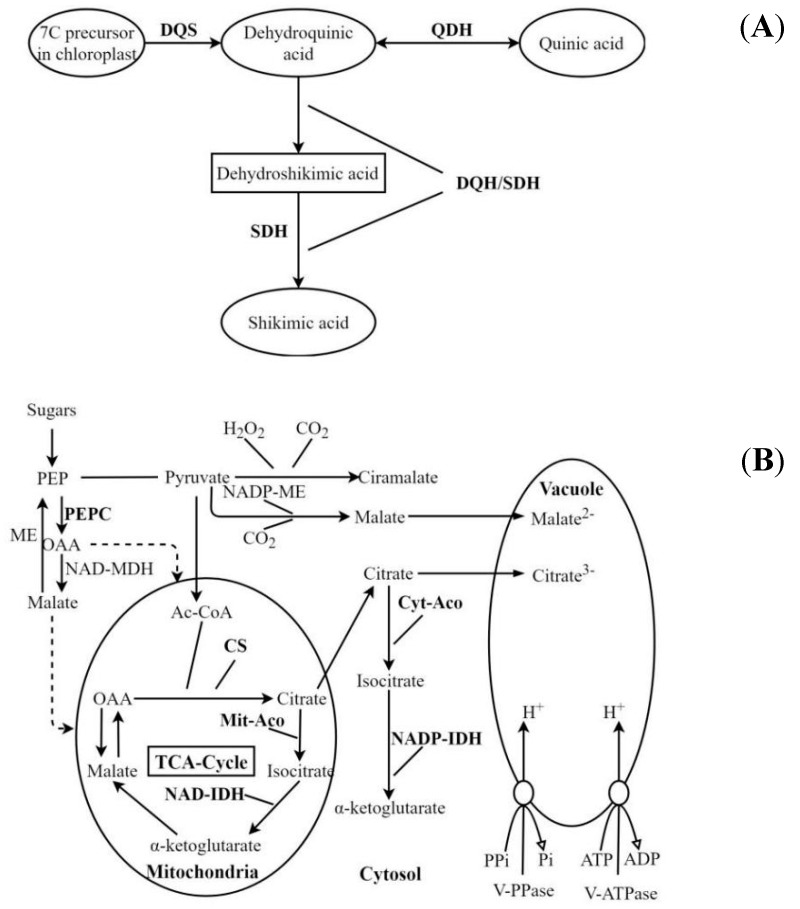
Schematic representation of the quinic acid (**A**) and citric acid (**B**) metabolic pathways in fruits. PEP: Phosphoenolpyruvate; PEPC: Phosphoenolpyruvate carboxylase; OAA: Oxaloacetate; ME: Malic enzyme; NAD-MDH: NAD-Malate dehydrogenase; NADP-ME: NADP-Malic enzyme; Ac-CoA: Acetyl coenzyme A; CS: Citrate synthase; Mit-Aco: Mitochondrial aconitase; NAD-IDH: NAD-Isocitrate dehydrogenase; Cyt-Aco: Cytoplasmic aconitase; NADP-IDH: NADP-Isocitrate dehydrogenase; TCA-Cycle: Tricarboxylic acid cycle.

The content of organic acids in fruits is closely related to the activities of related metabolic enzyme. Many studies about the organic acids components and related metabolic enzyme have been carried out in the fruits of apple [19], *Citrus* [20], pear [21], apricot [22], jujube [23], plum [24], etc. Only a few studies on organic acid metabolism have been considered in kiwifruit over the past decades. Quinic acid is a side product of the shikimate pathway (Figure 1A), which could be regulated by the activity of Quinate dehydrogenase (QDH) [16]. Shikimic acid accumulated through a bifunctional enzyme with the activities of both Dehydroquinate dehydratase (DQH) and Shikimate dehydrogenase (SDH) [25]. In the previous studies, the activities of QDH and SDH in kiwifruit of ‘Hayward’, ‘Hort16A’ and ‘E4I6’ were measured, the activity of QDH was highest in the early period and then decreased with fruit development [18]. The citric acid metabolic pathway is very complicated in fruits (Figure 1B), studies have found that the key enzymes in citric acid metabolism are Citrate synthase (CS), Mitochondrial aconitase (Mit-Aco), Cytoplasmic aconitase (Cyt-Aco), Isocitrate dehydrogenase (IDH), and Phosphoenolpyruvate carboxylase (PEPC) [26].

The molecular regulation of organic acids content in fruit often involves polygenes, which is extremely complicated in general [27]. A gene encoding QDH enzyme has not been cloned from plants [28]. In the previous studies, gene expression analysis of *SDH* and *DQS* (encoding dehydroquinate synthase) was performed in three kinds of species of *Actinidia*, which indicated that the *DQS* gene was highly expressed in the early period and then decreased [16]. Moreover, the trend in changes of the quinic acid content was consistent with the relative gene expression levels of *SDH* and *DQS* during fruit development [16]. On the other hand, the activities of NADP-IDH and Mit-Aco could be regulated by genes of *IDH* and *Aco*, respectively, during fruit development of *Citrus* [26,29,30]. Moreover, previous studies have shown that the content of citric acid is not related to the gene expression level of *CitCSs* and *CitPEPCs* during fruit development of *Citrus* [31,32]. However, the metabolism of organic acid in fruit is very complex, which is often regulated by a lot of functional genes, and needs more in-depth research.

In addition, there have been no studies that examined the changes of the organic acids components and content, the variations of related metabolic enzyme activities and relative gene expression levels during fruit development and maturation of *A. eriantha*. Therefore, our study selected the ‘Ganmi 6’ as materials, the components and content of organic acids were determined during fruit development and maturation with a high-performance liquid chromatography (HPLC) system. The activities of organic acid-metabolizing enzyme were analyzed by using ultraviolet spectrophotometry, gene expression levels of the major organic acid-metabolizing enzyme were detected and analyzed by quantitative real-time PCR (qRT-PCR). Our study provided new insight into the characteristics of organic acid metabolism in the fruit of *A. eriantha*, presented a theoretical basis for further study on their metabolism and regulation mechanism at a molecular level.

## 2. Results

### 2.1. Quinic Acid and Citric Acid Were the Main Organic Acids in the Fruit of ‘Ganmi 6’

**Figure 2:**
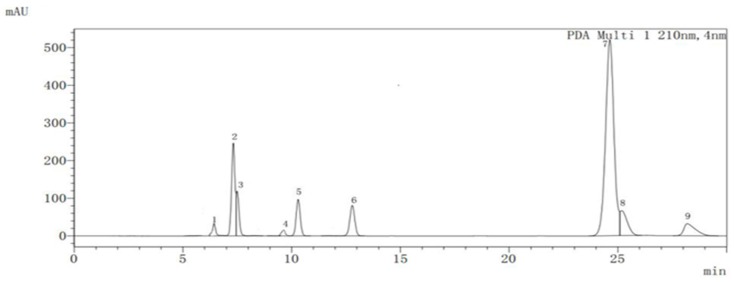
Chromatogram of organic acids in the fruit of *Actinidia eriantha* ‘Ganmi 6’ obtained at 210 nm. 1: Oxalic acid; 2: Tartaric acid; 3: Quinic acid; 4: Malic acid; 5: Lactic acid; 6: AsA; 7: Citric acid; 8: Fumaric acid; 9: Succinic acid.

The analysis of organic acids during fruit development and maturation of ‘Ganmi 6’ showed that eight kinds of organic acids (quinic acid, citric acid, malic acid, lactic acid, oxalic acid, succinic acid, tartaric acid, and fumaric acid) and AsA were detected (Figure 2).

**Figure 3:**
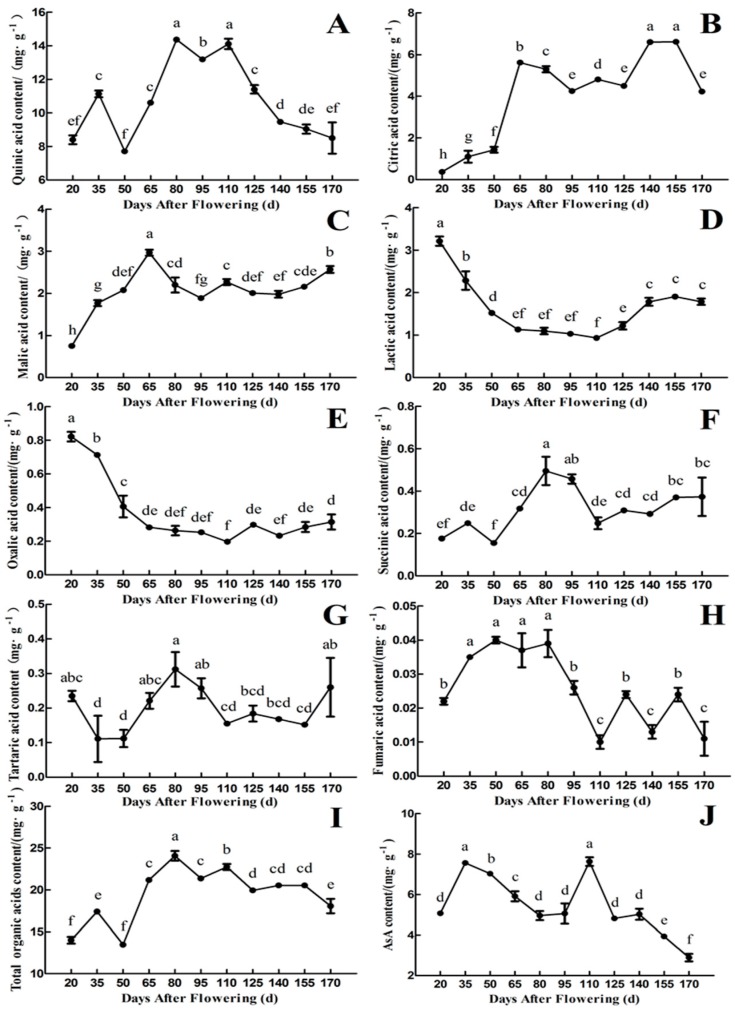
Changes in the components and content of organic acids and AsA during fruit development and maturation of *A. eriantha* ‘Ganmi 6’, including quinic acid (**A**), citric acid (**B**), malic acid (**C**), lactic acid (**D**), oxalic acid (**E**), succinic acid (**F**), tartaric acid (**G**), fumaric acid (**H**), total organic acids (**I**), and AsA (**J**). Data are means ± standard deviations of three biological repetitions. Error bars represent standard deviations of means. Different lowercase letters are significantly different at *p* < 0.01. Data were analyzed with two-way analysis of variance (ANOVA) and Duncan’s multiple range tests.

As shown in Figure 3, quinic acid and citric acid were the main organic acids in the fruit of ‘Ganmi 6’. The variation ranges were 7.71~14.37 and 0.37~6.61 mg·g^−1^ FW, respectively, accounted for 62.68~83.21% of the total organic acids during different fruit developmental periods. The content of quinic acid is higher than that of other organic acids during the whole developmental periods, and the trend in changes of quinic acid content was consistent with total organic acids content. (Figure 3A,I). During the early periods of fruit development, the content of quinic acid was increased continually from 20 to 80 Days After Flowering (DAF; except 50 DAF), and the highest quinic acid content was 14.37 mg·g^−1^ FW at 80 DAF. Then it decreased from 110 to 170 DAF with fruit development and maturation (Figure 3A).

The content of citric acid increased in the early periods (20~65 DAF), then decreased from 65 to 95 DAF, and increased again with fruit development (95~155 DAF, except 125 DAF). It reached a maximum value of 6.61 mg·g^−1^ FW at 155 DAF. Finally, it decreased rapidly until maturity (170 DAF) (Figure 3B). The trend in changes of malic acid content was similar to that of citric acid content, with the maximum value of 2.97 mg·g^−1^ FW at 65 DAF (Figure 3C). The content of lactic acid and oxalic acid showed similar trend, which reached a maximum value of 3.21 and 0.82 mg·g^−1^ FW, respectively, at 20 DAF. Then they decreased gradually before 110 DAF, followed by a small quantity of increasing with fruit development and maturation (110~170 DAF) (Figure 3D,E). The content of succinic acid and tartaric acid also showed similar trend, which reached a maximum value of 0.50 and 0.31 mg·g^−1^ FW, respectively, at 80 DAF (Figure 3F,G). The content of fumaric acid was extremely low during the whole fruit developmental periods, and the maximum value was only 0.04 mg·g^−1^ FW (Figure 3H).

The content of total organic acids reached a minimum value of 13.46 mg·g^−1^ FW at 50 DAF and a maximum value of 24.07 mg·g^−1^ FW at 80 DAF in the fruit of ‘Ganmi 6’. From 50 to 80 DAF, it accumulated at a high rate, the content of total organic acids decreased gradually after 80 DAF with fruit development and maturation (Figure 3I). The content of AsA was measured, as well. Interestingly, the trend in changes in AsA content was similar to that of quinic acid content, which reached a maximum value at 110 DAF (Figure 3J).

### 2.2. Analysis of Activities of Quinic Acid and Citric Acid-Related Enzyme

**Figure 4:**
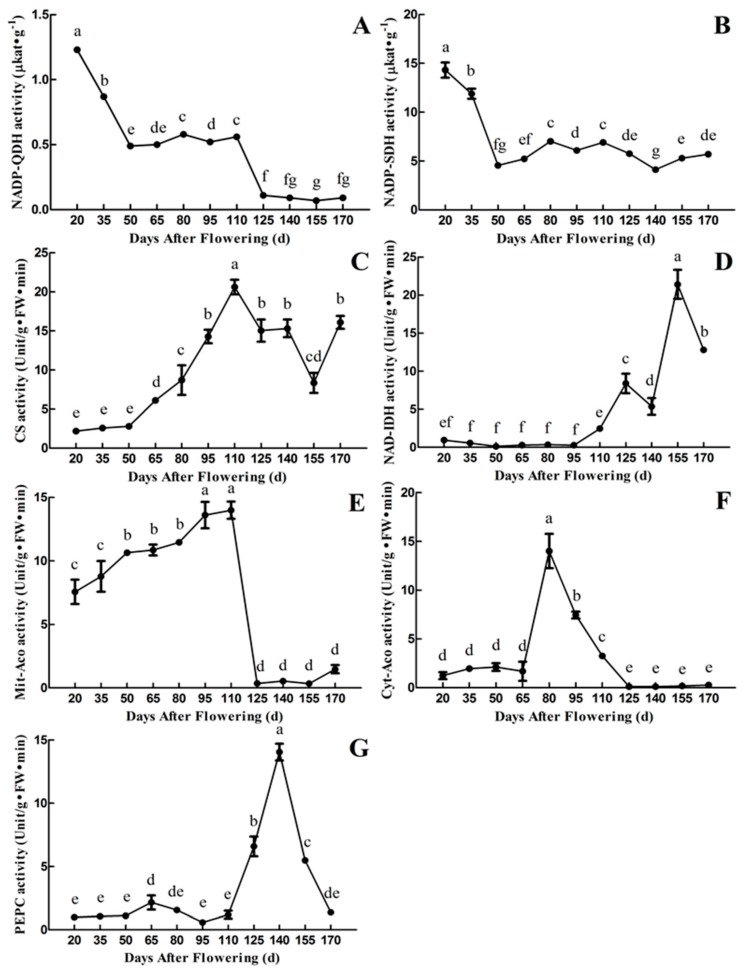
Changes in the activities of quinic acid and citric acid-related enzyme during fruit development and maturation of *A. eriantha* ‘Ganmi 6’, including NADP-QDH (**A**), NADP-SDH (**B**), CS (**C**), NAD-IDH (**D**), Mit-Aco (**E**), Cyt-Aco (**F**), and PEPC (**G**). FW: Fresh weight; NADP-QDH: NADP-Quinate dehydrogenase; NADP-SDH: NADP-Shikimate dehydrogenase; CS: Citrate synthase; NAD-IDH: NAD-Isocitrate dehydrogenase; Mit-Aco: Mitochondrial aconitase; Cyt-Aco: Cytoplasmic aconitase; PEPC: Phosphoenolpyruvate carboxylase. Data are means ± standard deviations of three biological repetitions. Error bars represent standard deviations of means. Different lowercase letters are significantly different at *p* < 0.01. Data were analyzed with two-way analysis of variance (ANOVA) and Duncan’s multiple range tests.

It can be seen from Figure 4A,B that the activities of NADP-Quinate dehydrogenase (NADP- QDH) and NADP-Shikimate dehydrogenase (NADP-SDH) are highest in the first period (20 DAF) and then decreased during fruit development and maturation, which is not consistent with the changes of quinic acid content. The activity of NADP-QDH reached a maximum level of 1.23 μkat·g^−1^ at 20 DAF, then decreased rapidly from 20 to 50 DAF. It maintained at a relatively stable level from 50 to 110 DAF, followed by a rapid decrease to the minimum value of 0.07 μkat·g^−1^ at 155 DAF. The activity of NADP-SDH reached a maximum level of 14.32 μkat·g^−1^ at 20 DAF, then decreased rapidly with fruit development and maturation. It remained at a relatively stable level from 50 to 110 DAF. In addition, the activity of NADP-SDH was about 10 times higher than that of the activity of NADP-QDH during fruit development and maturation.

CS catalyzes the condensation of Oxaloacetate (OAA) to Acetyl coenzyme A (AcCoA) to form citric acid in the tricarboxylic acid (TCA) cycle [29]. The activity of CS increased continuously from 20 to 110 DAF, reached the maximum level of 20.62 Unit·g^−1^ FW·min^−1^ at 110 DAF, then decreased to 8.35 Unit·g^−1^ FW·min^−1^ at 155 DAF, followed by a slight increase from 155 to 170 DAF. Overall, the trend in changes of CS activity is close to that of citric acid content in the fruit of ‘Ganmi 6’ (Figure 3B and Figure 4C).

The activity of NAD-Isocitrate dehydrogenase (NAD-IDH) was extremely low at early periods (20~110 DAF) and high in the later periods (125~170 DAF). The highest enzyme activity was 21.40 Unit·g^−1^ FW·min^−1^ at 155 DAF, followed by a rapid decrease from 155 to 170 DAF. From 125 to 170 DAF, the trend in changes of NAD-IDH activity is similar to that of citric acid content (Figure 3B and Figure 4D).

Aconitase (Aco) includes Mit-Aco and Cyt-Aco. Mit-Aco is involved in the metabolism of citric acid, catalyzes the formation of isocitrate from citric acid, and promotes the degradation and conversion of citric acid. While Cyt-Aco plays a role in a variety of biochemical processes [22]. The activity of Mit-Aco was higher than that of Cyt-Aco (except 80 DAF). It decreased and maintained at a low level after 110 DAF. The changes of Cyt-Aco activity showed a trend of “up-down-stable”. The highest activity was 14.00 Unit·g^−1^ FW·min^−1^ at 80 DAF, then decreased rapidly to a low level (125 DAF), finally remained at an extremely low activity until 170 DAF. However, the trend in changes of citric acid content is not consistent with Mit-Aco and Cyt-Aco activities (Figure 3B and Figure 4E,F).

OAA is the precursor for citric acid synthesis, which could be produced by Phosphoenolpyruvate (PEP) under the catalysis of PEPC. The activity of PEPC remained at a very low level, with small fluctuations from 20 to 110 DAF, then increased rapidly to the maximum value of 14.04 Unit·g^−1^ FW·min^−1^ at 140 DAF, followed by a rapid decrease to 1.37 Unit·g^−1^ FW·min^−1^ at 170 DAF. To some extent, the trend in changes of PEPC activity is affiliated with the citric acid content during the early (20~50 DAF) and late (125~170 DAF) periods (Figure 3B and Figure 4G).

### 2.3. Quantitative Real-Time PCR Analysis of Quinic Acid and Citric Acid Metabolism Enzyme Gene

**Figure 5:**
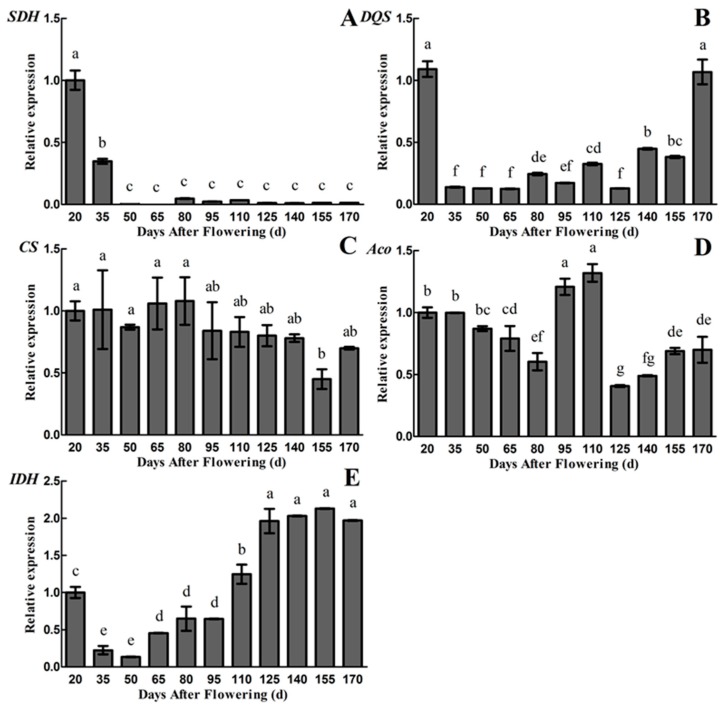
Changes in relative gene expression levels during fruit development and maturation of *A. eriantha* ‘Ganmi 6’, including *SDH* (**A**), *DQS* (**B**), *CS* (**C**), *Aco* (**D**) and *IDH* (**E**). Data are means ± standard deviations of three biological repetitions. Error bars represent standard deviations of means. Different lowercase letters are significantly different at *p* < 0.01. Data were analyzed with two-way analysis of variance (ANOVA) and Duncan’s multiple range tests.

Since the gene encoding NADP-QDH enzyme has not been cloned from plants, *DQS* gene has a similar function with *QDH*, we performed the gene expression analysis of *SDH* and *DQS* in the quinic acid metabolic pathway [28]. The results of qRT-PCR using primers of *SDH* and *DQS* are shown in Figure 5A,B. The metabolic mechanism of quinic acid become clearer, based on the results. The results showed that the *SDH* gene is highly expressed in the early periods (20~35 DAF), and the relative gene expression level is extremely low or even not expressed from 50 to 170 DAF, which is similar to the changes of NADP-SDH activity (Figure 4B and Figure 5A). The relative gene expression level of *DQS* was high at 20 DAF and was low from 35 to 125 DAF, followed by an increase to 1.07 at 170 DAF. However, the trend in changes of *DQS* gene expression level is not consistent with the NADP-QDH or NADP-SDH activity, as well as the quinic acid content (Figure 3A, Figure 4A,B and Figure 5B).

In addition, qRT-PCR analysis of citric acid metabolism enzyme gene (*CS*, *Aco* and *IDH*) have been conducted. The highest relative gene expression level of *CS* was observed during the whole fruit developmental periods (except 155 DAF) of ‘Ganmi 6’ (Figure 5C). The results are not exactly consistent with the changes of citric acid content and CS activity (Figure 3B, Figure 4C and Figure 5C). The relative gene expression level of *Aco* decreased slightly from 20 to 80 DAF, and then increased rapidly to reach the maximum value of 1.32 at 110 DAF, followed by a rapid decrease to reach the minimum value of 0.41 at 125 DAF and a slight increase from 125 to 170 DAF (Figure 5D). At the same time, the activity of Mit-Aco also reached a maximum value at 110 DAF. The trend in changes of *Aco* gene expression level is similar to Mit-Aco activity, rather than Cyt-Aco activity, but the similarity with citric acid content is not high (Figure 3B, Figure 4E,F and Figure 5D). The relative gene expression level of *IDH* decreased rapidly to reach the minimum value of 0.13 from 20 to 50 DAF, and then increased gradually to reach the maximum value of 2.13 at 155 DAF, which is partly consistent with citric acid content and NAD-IDH activity (Figure 3B, Figure 4D and Figure 5E).

### 2.4. Correlation Analysis of Major Organic Acids Content, Related Enzyme Activities and Gene Expression

The correlation between gene expression, enzyme activities and organic acids content in developing fruit of ‘Ganmi 6’ was analyzed (Table 1). Significant correlations were apparent between the content of total organic acids and quinic acid or citric acid in the fruit of ‘Ganmi 6’. The correlations between the activities of NADP-QDH and NADP-SDH and the content of quinic acid were not significant. Furthermore, no significant correlations were found between the content of citric acid and the activities of CS, NAD-IDH, Mit-Aco, Cyt-Aco and PEPC. However, some positively or negatively significant correlations were discovered between different kinds of enzyme activities.

In the fruit of ‘Ganmi 6’, the relative gene expression level of *SDH* was significantly positively correlated with the activities of NADP-QDH and NADP-SDH. However, the correlations between the relative gene expression level of *SDH* and the content of quinic acid or total organic acids was not significant. Moreover, the correlations between the relative gene expression level of *DQS* and other data were not significant. The relative gene expression level of *CS* was significantly positively correlated with the activity of Mit-Aco and significantly negatively correlated with the activity of NAD-IDH, but its correlations with citric acid content and CS activity were not significant. The relative gene expression level of *Aco* was significantly positively correlated with the activity of Mit-Aco and significantly negatively correlated with the activity of PEPC. The relative gene expression level of *IDH* was significantly positively correlated with the activities of NAD-IDH and PEPC, while it was significantly negatively correlated with the activity of Mit-Aco and the relative gene expression level of *CS*.

## 3. Discussion

The organic acids content in fruits is one of the important indicators affecting fruit quality. Fruit quality is also affected by the acid-sugar ratio, while the acid-sugar ratio is mainly determined by acids content [21]. In this study, we reported the organic acids accumulation during fruit development and maturation of *A. eriantha*. Eight kinds of organic acids and AsA were detected in the fruit of ‘Ganmi 6’, the content of quinic acid and citric acid accounted for 43.99~63.99% and 2.65~32.19% of the total organic acids content, respectively. However, only four kinds of organic acids, including quinic acid, citric acid, malic acid and tartaric acid were detected in three kinds of kiwifruit in previous studies [14], which may be due to the difference in organic acids components and content in different kinds of kiwifruit. The content of organic acids accumulated continuously during fruit development, and then decreased during the maturation, which is consistent with the previous studies on pear, jujube, apricot and *Prunus mume* [21,23,33,34]. Correlation analysis showed that the content of quinic acid and citric acid were significantly correlated with the content of total organic acids, which is consistent with the results on *A. chinensis, A. deliciosa and A. arguta* [16]. These results suggested that quinic acid and citric acid are the main organic acids in the fruit of ‘Ganmi 6’. However, the content of quinic acid in the fruit of ‘Ganmi 6’ was lower than *A. chinensis* and *A. deliciosa* at the same period [16], which may be due to the diversity of different species in kiwifruit and different environmental conditions of the growing area.

In the shikimate pathway, dehydroquinic acid produces quinic acid-catalyzed by QDH. While shikimic acid could also be produced by dehydroquinic acid-catalyzed a bifunctional enzyme with the activities of both DQH and SDH in plants [25]. In this study, the changes of quinic acid content were not exactly related to the activities of NADP-QDH and NADP-SDH, which is partly consistent with the previous study on *A. chinensis* [16]. Quinic acid content and NADP-QDH activity decreased gradually from 110 to 170 DAF, while NADP-SDH activity still maintained at a medium level, which may indicate that quinic acid could be used to make shikimic acid with fruit development and maturation. The results are consistent with the previous study [16]. Citric acid is an intermediate in the TCA cycle, and the synthesis and degradation of citric acid is closely related to the TCA cycle [35]. In our study, the changes of citric acid content were partly consistent with the activities of CS, NAD-IDH and PEPC, but not consistent with the activities of Mit-Aco and Cyt-Aco. These results may indicate that CS, NAD-IDH and PEPC are important enzymes which affected the citric acid metabolism, which is partly consistent with the previous studies [22,36]. Several studies reported that Aco and NAD-IDH promoted the decomposition of citric acid [37,38]. In this study, there was no contrary trends and correlations between the content of citric acid and the activities of NAD-IDH, Mit-Aco and Cyt-Aco. The results are similar to those found in studies on pear [39]. In a word, the activities of CS, NAD-IDH and PEPC may play important roles in the citric acid accumulation during fruit development and maturation of ‘Ganmi 6’. However, the accumulation of organic acid in the fruit of *A. eriantha* was regulated by multiple enzymes, rather than a single enzyme, and the specific molecular mechanism needs further study.

Nowadays, there is a wide range of reports about the mechanism of organic acid metabolism during fruit development and maturation [26,30,31,40,41,42,43,44]. The shikimate pathway is an important metabolic pathway present in plants, and is the main bridge connecting sugar metabolism and secondary metabolism [28]. QDH and SDH enzymes are important in the quinic acid metabolic pathway. The gene encoding QDH enzyme has not been cloned from plants, so we mainly concentrated on the studies of *SDH* and *DQS* genes. Besides, previous studies have shown that the *DQS* gene may have an indirect effect on the biosynthesis of quinic acid [16]. In this study, the changes of the relative gene expression level of *SDH* or *DQS* is consistent with the activities of NADP-QDH and NADP-SDH, but not exactly consistent with the content of quinic acid at early periods (20~35 DAF) of fruit development. The relative gene expression level of *SDH* was significantly positively correlated with the activities of NADP-QDH and NADP-SDH, which is consistent with the results on *Citrus* [16,40]. These results suggested that quinic acid may be synthesized at the early periods of fruit development and the activities of NADP-QDH and NADP-SDH may be regulated by *SDH* gene, *DQS* gene did not have a decisive effect on the quinic acid accumulation in the fruit of ‘Ganmi 6’.

In the citric acid metabolic pathway, the changes of citric acid content were consistent with the relative gene expression level of *IDH* in general. However, there were no significant correlations between the content of citric acid and the relative gene expression levels of *CS* and *Aco*, which are consistent with the study on *Citrus* [31,32]. In addition, the trend in changes of *Aco* gene expression level is similar to Mit-Aco activity, rather than Cyt-Aco activity. Moreover, the trend in the changes of *IDH* gene expression level is similar to NAD-IDH activity, which is consistent with the results of correlation analysis. However, there was no consistency between the relative gene expression level of *CS* and the activity of CS, which is consistent with the previous study on *Citrus grandis* [45]. Finally, there are negatively significant correlations between the relative gene expression level of *CS* and the activity of NAD-IDH, as well as the relative gene expression level of *IDH.* These results indicated that the relative gene expression level of *IDH* may be the main reasons for regulating the changes of the content of citric acid and the activities of Mit-Aco and NAD-IDH may be regulated by *Aco* and *IDH* gene, respectively, during fruit development and maturation of ‘Ganmi 6’.

## 4. Materials and Methods

### 4.1. Plant Materials

**Figure 6:**
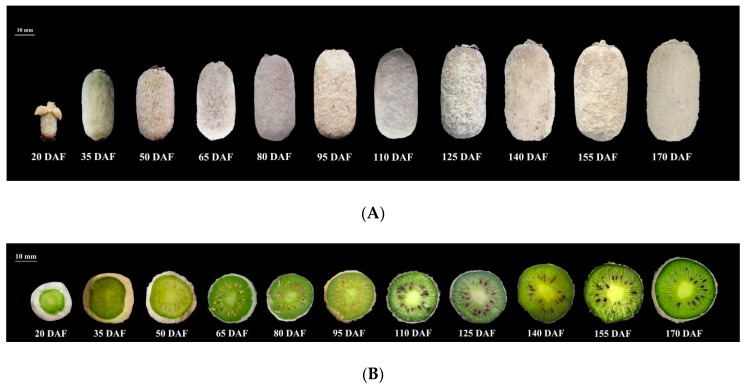
Fruits of *A. eriantha* ‘Ganmi 6’ at different developmental periods. (**A**) fruits; (**B**) cross sections. DAF: Days After Flowering.

The fruits of *A. eriantha* ‘Ganmi 6’ used in this study were harvested from the Germplasm Resources Nursery of Institute of Kiwifruit, Fengxin County, Jiangxi Province, China (28°70’ N, 115°38’ E, elevation, 75 m). The fruit samples were collected at eleven developmental periods (20, 35, 50, 65, 80, 95, 110, 125, 140, 155 and 170 DAF) from 31 May 2017 to 28 October 2017 (Figure 6). 15~20 fruits were collected each time, and the fruit samples were put into ice boxes. Then, they were transported to the laboratory, the outer pericarp was cut into small sections, before being immediately frozen in liquid nitrogen, and stored at −80℃ for further analysis.

### 4.2. Extraction and Determination of Organic Acids

The extraction and determination of organic acids were conducted according to a previous protocol with minor modifications [14]. The extract was used to measure organic acids content using an HPLC system (Shimadzu, Kyoto, Japan). The injection volume was 20 µL, detection wavelength was 210 nm. An Shim-pack GISS C18 column (5 µm, 4.6 × 250 mm) was used to conduct chromatographic separation, column temperature remained at 30 °C. The mobile phase was 0.01 mol/L H_2_SO_4_ solution (pH 2.6), with a flow rate of 0.5 mL/min. Three replicates of each sample were analyzed.

### 4.3. Determination of Enzyme Activity

Enzyme samples of NADP-QDH and NADP-SDH were prepared as previously described [46]. The NADP-QDH activity was determined in the reaction mixture containing glycine buffer (0.2 mM, pH 10.0), 2 mM Nicotinamide adenine dinucleotide phosphate (NADP) and 25 mM quinic acid. The NADP-SDH activity was determined in the reaction mixture containing glycine buffer (0.2 mM, pH 10.0), 2 mM NADP and 10 mM shikimic acid.

Enzyme samples of NAD-IDH, CS, PEPC, and Aco were prepared and determined with minor modifications as previously described [20,47]. The reaction volume was changed to 3 mL. NAD-IDH activity was determined in the reaction mixture containing 300 μL Hepes (40 mM, pH 8.2), 150 μL Nicotinamide adenine dinucleotide (NAD; 800 pM), 150 μL MnSO_4_ (200 μM), 1050 μL ddH_2_O, 150 μL Sodium isocitrate (2 mM), and 1200 μL enzyme extract. CS activity was determined in the reaction mixture containing 300 μL Tris-HCl (40 mM, pH 9.0), 150 μL 5,5’-Dithiobis-(2-nitrobenzoic acid) (DTNP, 40 μM), 150 μL AcCoA (40 μM), 300 μL ddH_2_O, 1500 μL OAA (4 mM), and 600 μL enzyme extract. PEPC activity was determined in the reaction mixture containing 150 μL Tris-HCl (40 mM, pH 8.5), 150 μL MgCl_2_ (2 mM), 150 μL KHCO_3_ (10 mM), 150 μL Glutathione (GSH; 500 μM), 150 μL Nicotinamide adenine dinucleotide (NADH; 150 μM), 150 μL ddH_2_O, 1500 μL PEP (2 mM), and 100 μL enzyme extract. Aco activity was determined in the reaction mixture containing 300 μL Tris-HCl (40 mM, pH 7.5), 150 μL NaCl (100 μM), 1200 μL ddH_2_O, 450 μL Aconitic acid (200 μM), and 1200 μL enzyme extract. All enzyme activities were determined by using a UV-2600 spectrophotometer (Shimadzu, Kyoto, Japan). Three replicates of each sample were analyzed.

### 4.4. Total RNA Extraction and Quantitative Real-Time PCR Analysis

Total RNA was extracted through a Quick RNA isolation Kit (Huayueyang Biotechnology, Beijing, China). RNA quality was evaluated by agarose gel electrophoresis and using the NanoDrop system (Implen, Los Angeles, CA, USA). cDNA was synthesized with the PrimeScript™ RT reagent Kit with gDNA Eraser (Perfect Real Time) (TaKaRa, Inc., Dalian, China).

Full-length sequences of genes, including *CS*, *Aco*, *IDH*, *SDH* and *DQS* were searched in kiwifruit gene database, and qRT-PCR primers were designed by Primer 5.0 software, according to them [48]. The qRT-PCR primers were synthesized by Sangon Biotechnology, Shanghai, China (Table 2). The qRT-PCR mixture (20 μL) contained 10 μL TB Green™ Premix Ex Taq™ (Tli RNaseH Plus) (TaKaRa, Inc., Dalian, China), 0.5 μL of the forward and reverse primers for each gene, 1 μL cDNA template, and 8 μL ddH_2_O. The LightCycler^®^ 480 Real-Time PCR System (Applied Biosystems, Waltham, MA, USA) was used to conduct the reaction. The conditions for the qRT-PCR amplifications were as follows—95 °C for 5 min, followed by 40 cycles of 5 s at 95 °C 30 s at 60 °C and 20 s at 72 °C. The *β-actin* in the kiwifruit was considered as the reference gene for normalization [49]. All analyses were repeated three times using biological replicates. The relative expression levels were calculated using the 2^−ΔΔCT^ method [50].

### 4.5. Statistical Analysis

All assays were performed with three biological replicates. Data were presented as means ± standard deviations. GraphPad Prism 5 (GraphPad Software Inc., San Diego, CA, USA) was used for chart preparation. Two-way analysis of variance (ANOVA) and Duncan’s multiple range tests was carried out using the SPSS 22.0 software (SPSS Inc., New York, NY, USA). Pearson’s correlation was calculated to describe associations between selected variables. Unless otherwise stated, differences were significant at *p* < 0.01.

## 5. Conclusions

In conclusion, our results suggested that quinic acid and citric acid are the main organic acids in the fruit of ‘Ganmi 6’. CS, NAD-IDH, and PEPC may be involved in regulating citric acid biosynthesis in the fruit of ‘Ganmi 6’. Furthermore, the accumulation of organic acid in the fruit of *A. eriantha* was regulated by the combination of multiple enzymes. At the same time, the activities of NADP-SDH, Mit-Aco, and NAD-IDH was regulated by related genes of *SDH*, *Aco* and *IDH*, respectively, at the transcriptional level. However, the activity of CS was not regulated by the *CS* gene at the transcriptional level, and it may be affected by post-translational modification. *DQS* gene did not have a decisive effect on the accumulation of quinic acid in the fruit of ‘Ganmi 6’. Our results provided new insight into the characteristics of organic acid metabolism in the fruit of *A. eriantha*. In the future, more research should be conducted in order to broaden the understanding of the molecular mechanisms involved in those biosynthetic pathways in *A. eriantha*.

## Figures and Tables

**Table 1:** The correlations in major organic acids content, enzyme activities and relative gene expression levels during developing fruit of *A. eriantha* ‘Ganmi 6’.

	Quinic	Citric	Total	QDH	SDH	CS	IDH	Mit-Aco	Cyt-Aco	PEPC	e*SDH*	e*DQS*	e*CS*	e*Aco*	e*IDH*
Quinic	1.000														
Citric	0.295	1.000													
Total	0.792 **	0.805 **	1.000												
QDH	0.107	−0.755 **	−0.376	1.000											
SDH	−0.019	−0.729 *	−0.411	0.863 **	1.000										
CS	0.432	0.601	0.602	−0.582	−0.493	1.000									
IDH	−0.355	0.473	0.096	−0.674 *	−0.319	0.265	1.000								
Mit-Aco	0.550	−0.303	0.133	0.614 *	0.204	−0.151	−0.771 **	1.000							
Cyt-Aco	0.711 *	0.089	0.498	0.254	0.021	−0.005	−0.443	0.603 *	1.000						
PEPC	−0.206	0.553	0.193	−0.580	−0.416	0.312	0.347	−0.682 *	−0.367	1.000					
e*SDH*	−0.266	−0.698 *	−0.558	0.809 **	0.922 **	−0.521	−0.258	0.068	−0.121	−0.254	1.000				
e*DQS*	−0.465	−0.220	−0.374	0.155	0.392	0.045	0.280	−0.347	−0.277	−0.052	0.559	1.000			
e*CS*	0.334	−0.436	−0.040	0.701 *	0.447	−0.407	−0.896 **	0.627 *	0.469	−0.377	0.356	−0.175	1.000		
e*Aco*	0.295	−0.411	−0.087	0.590	0.387	−0.005	−0.420	0.756 **	0.149	−0.647 *	0.274	0.000	0.190	1.000	
e*IDH*	−0.202	0.565	0.226	−0.681 *	−0.314	0.602	0.807 **	−0.824 **	−0.446	0.640 *	−0.185	0.410	−0.752 **	−0.501	1.000

Note: ** Significant correlation at the 0.01 level (bilateral), * Significant correlation at the 0.05 level (bilateral). Quinic: Quinic acid content; Citric: Citric acid content; Total: Total organic acids content; QDH: NADP-Quinate dehydrogenase activity; SDH: NADP-Shikimate dehydrogenase activity; CS: Citrate synthase activity; IDH: NAD-Isocitrate dehydrogenase activity; Mit-Aco, Mitochondrial Aconitase activity; Cyt-Aco, Cytoplasmic Aconitase activity; PEPC, Phosphoenolpyruvate carboxylase activity; e*SDH*: Relative gene expression level of *SDH*; e*DQS*: Relative gene expression level of *DQS*; e*CS:* Relative gene expression level of *CS*; e*Aco:* Relative gene expression level of *Aco*; e*IDH:* Relative gene expression level of *IDH*.

**Table 2:** Primers used for quantitative real-time PCR.

Gene	Accession No.	Forward primer (5’-3’)	Reverse primer (5’-3’)
*CS*	Achn032171	AGGTTGAGATGGGAGGATG	GAAGGTAGCGGTATCATCG
*Aco*	Achn079831	CGCTCAGTATTAGGGCTCA	GTTCATAGCATCCCGCATA
*IDH*	Achn019991	GAGCGATACGAAGTCCACG	TCTTGGGTGGTGCTGTCTC
*SDH*	Achn219731	GAAGGTGGTCAATACGA	TCACCCATAGTTACCTCA
*DQS*	Achn075801	GAAGGTGGTCAATACGA	TGTTGAAGGCTATGTAAAG
*β-actin*	Achn107181	GCTTACAGAGGCACCACTCAACC	CCGGAATCCAGCACCAATACCAG
